# Enhancing Carbon Fiber-Reinforced Polymers’ Performance and Reparability Through Core–Shell Rubber Modification and Patch Repair Techniques

**DOI:** 10.3390/polym17030407

**Published:** 2025-02-03

**Authors:** Dionisis Semitekolos, Sofia Terzopoulou, Costas Charitidis

**Affiliations:** RNANO Lab—Research Lab of Advanced, Composite, Nanomaterials and Nanotechnology, School of Chemical Engineering, National Technical University of Athens, 9 Heroon Polytechneiou st., Zografos, GR-15773 Athens, Greece

**Keywords:** CFRPs, core–shell rubber, fracture toughness, impact modifiers, patch repair

## Abstract

Carbon fiber-reinforced polymers (CFRPs) are widely used in high-performance applications, but their inherent brittleness and susceptibility to impact damage remain critical challenges. This study investigated the effect of core–shell rubber (CSR) particles as impact modifiers on the mechanical properties of CFRPs and evaluated patch repair techniques for damaged CFRP panels. Mechanical tests, including flexural, tensile, short-beam, fracture toughness, and impact tests, were conducted on reference and CSR-modified specimens to assess their structural performance. The CSR-modified samples demonstrated significant improvements in energy absorption and fracture toughness, with a 50% increase in impact strength and up to 181% improvement in absorbed energy during Mode I fracture testing. However, slight reductions in flexural and tensile strengths were observed due to the softening effect of CSR particles. Fracture surface analysis revealed distinct failure mechanisms, with Scanning Electron Microscopy imaging showing consistent fiber pull-out behavior in tensile and flexural tests, but more stable delamination propagation in CSR-modified specimens during short-beam shear tests. Patch repair effectiveness was assessed through drop-weight impact tests on damaged panels repaired with patches containing CSRs of two thicknesses. Patches of equal thickness to the damaged panel successfully restored structural integrity and enhanced energy absorption by 37% compared with the reference samples, while thinner patches (as a suggestion to reduce production costs) failed to withstand impact loads effectively. Non-destructive testing (NDT) via ultrasonic C-scans confirmed reduced delamination and damage depth in CSR-modified repaired panels, validating the toughening effect of CSR particles. These findings demonstrate the potential of CSR-modified resins to improve CFRPs’ performance and provide effective repair solutions for extending the service life of damaged composite structures, rendering them especially suitable for applications demanding high damage tolerance and durability, including aerospace structures, automotive body panels, and energy-absorbing crash components.

## 1. Introduction

The technological demands of the contemporary engineering sector are continuously growing, creating the requirement for advanced materials with exceptional properties. Composite materials, and more specifically carbon fiber-reinforced polymers (CFRPs), are gaining significant attention in several industries, such as aerospace, automotive, wind energy, leisure, and sports, due to their unique attributes related to their chemical, physical, and mechanical performance compared with other conventional materials. CFRPs consist of two constituents: carbon fibers and a polymer matrix. The combination of these creates a material that possesses characteristics such as high stiffness and corrosion and fatigue resistance, and, most importantly, the resultant structure is conveniently lightweight [1].

Carbon fibers (CFs) are included among the most attractive reinforcing materials used in the manufacture of fiber-reinforced polymers (FRPs). They are mainly manufactured through pyrolysis, a thermal processing of organic precursor fibers derived from rayon, polyacrylonitrile (PAN), or mesophase pitch, with PAN-based fibers comprising the primary chemical precursor for the manufacture of commercial high-quality CFs. Among several properties that distinguish this kind of precursor from the others, the elevated carbon yield, high tensile strength, and quick processing time appear to be the most worth mentioning [2]. There are also other types of fibers, such as aramid, boron, basalt, and even naturally originating, but their use is limited due to their lower performance [3]. Generally, CFs can be fabricated into various forms, including fabric, woven, yarn, or chopped fibers, while the most typical matrix used in CFRP manufacturing is thermoset polymers, without excluding, however, the possibility of using thermoplastics [1,4]. The principal role of the matrix is to offer stability against compression, enhancing CFRPs’ structures, so as to gain the potential to be exposed to extreme conditions [5,6].

Nevertheless, due to the involvement of CFRPs in high-performance applications, their production is constantly increasing, leading to an enlarged amount of waste. Traditionally, efforts to manage this waste have focused on recycling end-of-life (EoL) CFRP parts, aiming to recover valuable fibers for reuse. However, an equally important approach that has received less attention in the literature is the extension of the service life of CFRPs, which could significantly reduce waste generation and the demand for virgin raw materials. This study addresses this gap by investigating the incorporation of CSR particles into CFRPs in a high concentration (25% *w*/*w* in resin Part A). The addition of CSR particles aims to enhance the mechanical performance and durability of CFRPs, particularly their impact resistance and energy absorption, thereby extending their operational lifespan. By focusing on service life extension, this study provides an alternative for promoting sustainability in CFRP applications [7,8].

EoL CFRPs’ disposal usually takes place in landfills or through incineration, ordinary and affordable procedures that are, however, characterized by their relatively negative environmental impact. Precisely, landfills possess large surfaces of land, contaminating ground water, while during incineration, detrimental gases are generated. Consequently, the application of recycling methods for the exploitation of EoL CFRP parts appears to be particularly crucial [8]. The main processes that are already being examined and evaluated include mechanical, thermal, and chemical recycling. Mechanical recycling involves grinding the composite parts into smaller pieces that can be reused as fillers or reinforcers in bulk or sheet molding compounds and others. Concerning thermal recycling, high temperatures are reached to achieve the decomposition of the matrix and retrieve undamaged CFs of high quality. The most widely used method is pyrolysis, while fluidized bed technology is also an applicable technique. Regarding chemical recycling, solvents in a sub- or supercritical state are utilized, reclaiming CFs with high potential. When employing the abovementioned techniques, a life cycle of CFRPs is formed, effectively decreasing the environmental footprint arising from the disposal of wastes [8,9].

Another applicable alternative that could contribute to the reduction of CFRPs’ waste is the prolongation of service life of CFRPs that can occur when introducing core–shell rubbers (CSR) in the fiber–matrix composite, usually containing epoxy resin. Even though this type of resin is characterized by enhanced mechanical properties, such as high tensile strength and Young’s modulus, the existing of crosslinking explains the high level of brittleness, as well as the insufficient resistance to crack initiation [10]. This technique refers to the alteration of epoxy resin so as to enhance the ability of composite laminates to withstand impact, which can be accomplished by reinforcing the resin. Epoxy toughening can occur when using carboxyl-terminated butadiene acrylonitrile (CTBN), CSR, and/or silica nanoparticles. Furthermore, by introducing both silica nanoparticles and rubber particles, the impact and tensile properties are concurrently upgraded.

The focus of the present study was to improve the impact strength of a polymer matrix FRP by incorporating CSR, with the ultimate aim of extending the service life of the resulting composite materials and making them more resistant to impact damage. The introduction of those additives affects the impact behavior of CFRPs through three mechanisms; firstly, by the energy absorption that takes place due to the ability of the rubber phase to deform with the delivered impact energy. Secondly, during impact, the elastomeric core of the CSR particles can undergo localized cavitation. This process involves the formation of micro-voids within the rubber core, which dissipates a portion of the impact energy and helps to relieve stress concentrations in the surrounding matrix. By dispersing the impact energy in this manner, the material’s resistance to impact damage is improved. Lastly, these particles have the potential to absorb the vibrations and terminate the propagation of cracks [11].

**Figure 1:**
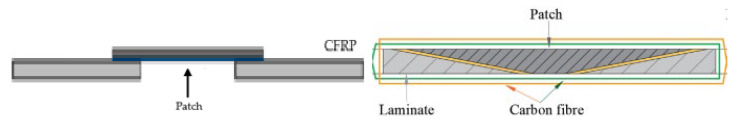
Configuration of patch (**left**) and scarf repair (**right**) on damaged CFRPs [14,15].

Additionally, patch repair was investigated as a technique of restoring impaired CFRPs. Specifically, this method refers to the repair of a damaged CFRP, which might also have reached EoL, by using patches of the same material as the pristine composite panel. There are two main types of layout: patch repair and scarf repair (Figure 1). Patch repair includes the adhesive bonding of a single- or double-sided patch on the damaged area of a CFRP panel; however, the latter type is not always applicable. Concerning scarf repair, the shape of the repair part completely suits the shape of the damage. Even though it is a much more effective method of repair, it also appears to be more challenging, due to the fact that if a patch of significant size is needed, then a large part of the undamaged composite material has to be detached [12]. Regarding the shape of the patches, several geometries have been examined, with the most appropriate, as to impact performance, being the circular one [13]. Other parameters that might vary are the location of the damage and the patch’s diameter and thickness [12].

Finally, CFRPs are usually subjected to impact loads during procedures related to operation or maintenance. The most common impairments include matrix cracking, fiber–matrix interfacial demolding, fiber breakage, or delaminations, with the latter being the most crucial among them. The consequence of these damages is the diminishing of the composite’s mechanical properties, limiting the potential of it. It is thus of significant importance to define the possible damage occurring in composite laminates, a process that is preferable to be realized using non-destructive testing. This method of testing encompasses ultrasonic C-scan imaging, which is a technique that can detect internal defects in the surrounding area of the damage to CFRPs caused by impact [16]. Specifically concerning the research that follows, impacted specimens that had previously been repaired by a patch repair procedure were subjected to the described type of testing, with the aim of the former analysis pertaining to the effectiveness of patch repair on composite panels.

To sum up, the investigation presented below included the incorporation of CSR nanoparticles in the matrix used for the manufacture of CFRPs, in order to define whether this addition enhances the mechanical properties of composites. Furthermore, the same system was used to create patches for artificially damaged CFRPs, with the aim of determining the possibility of repairing impaired or EoL CFRPs that, ultimately, possess equally remarkable or even improved properties. Finally, the effectiveness of those patches was tested with drop-weight impact tests, as well as ultrasonic scanning.

## 2. Materials and Methods

### 2.1. Materials

For the study that follows, two different kinds of fabric were chosen, in accordance with the mechanical test that would be conducted: G0926 from Hexcel Industries Inc., Casa Grande, AZ, USA, which is multiaxial, and C415 from Fibremax Composites, Volos, Greece, which is unidirectional. Their characteristics are presented in Table 1.

Two different types of matrices were utilized in this study, both comprising the same three-component resin system, with their compositions detailed in Table 2. The primary difference between these systems is the addition of CSR particles in System 2 at 25% *w*/*w*. These particles feature a polybutadiene rubber core encased by a hard, glassy shell with an average diameter of 100 nm. All components were supplied by Huntsman Corporation (The Woodlands, TX, USA), except for Component A of System 2, which was provided by Kaneka (Brussels, Belgium). Curing was performed for 4 h at 80 °C, followed by a post-curing step for an additional 4 h at 120 °C.

### 2.2. Composite Manufacturing

Two main investigations were performed within this study. The first focused on assessing the impact of core–shell rubbers on the most common mechanical properties of CFRPs. To this end, mechanical tests were conducted (including flexural, short-beam, tensile, impact, and fracture toughness) on reference panels and panels with impact modifiers (CSR). The second investigation evaluated different patch repair thicknesses using reference samples and samples repaired with patches made with MX156. The goal was to determine how the thickness of repair patches and the addition of CSR affected the integrity of the repaired CFRPs. Composites were fabricated using vacuum-assisted resin transfer molding (VA-RTM) (Supplementary file Figure S1).

#### 2.2.1. CSR Investigation

The composites used in this study incorporated two types of fabrics (as suggested by each standard): woven G0926 and unidirectional UD-C415, with nominal weights of 375 g/m^2^ and 415 g/m^2^, respectively. As a rule of thumb, each layer of G0926 and UD-C415 added approximately 0.375 mm and 0.415 mm, respectively, to the composite’s final thickness. The number of fabric layers in each composite was calculated on the basis of the fabric’s nominal weight and the target composite thickness, adhering to ASTM standards for each specific test. The following layers were used for each of the selected tests:

- Bending and tensile tests: 8 layers of G0926;
- Shear and fracture toughness tests: 12 layers of UD-C415;
- Impact tests: 10 layers of UD-C415.

For comparison, specimens were prepared with both pristine resin and MX 156. The fiber content in each composite panel was calculated using the following formula

(1) $$V_{r}\left(\%\right)=\frac{A_{r}\times N_{p}\times 0.1}{\rho_{r}\times h}$$

where

A_r_: Nominal weight of the fabric (g/m^2^);

N_p_: Number of layers in the composite;

*ρ*_r_: Density of the fabric (g/cm^3^);

h: Sample thickness (mm).

Fiber content values were calculated for reference panels as follows.

- Bending and tensile specimens: Average thickness of 3.05 mm, G0926 fabric with a nominal weight of 375 g/m^2^ and a density of 1.76 g/cm^3^, yielding 55.89% fiber content;
- Shear and fracture toughness specimens: Average thickness of 4.5 mm, 12 layers of UD-C415 fabric with a nominal weight of 415 g/m^2^ and a density of 1.82 g/cm^3^, resulting in 60.8% fiber content;
- Impact specimens: Average thickness of 4 mm, 10 layers of UD-C415 fabric, giving a fiber content of 56.5%.

The same methodology was applied for the MX156 panels. A summary of the panel properties and mechanical tests is provided below (Table 3). The similarity in fiber content between the reference and MX 156 panels ensured that the observed mechanical properties can be attributed solely to the matrix modification and not to variations in reinforcement.

#### 2.2.2. Patch Repair Mechanisms

For the second investigation, composite specimens were manufactured and tested for their impact behavior according to ASTM 7136 [17]. The target thickness for the specimens was 5 mm, and the dimensions were 150 mm × 100 mm. Each specimen was made using 12 plies of G0926 fabric (375 g/m^2^ nominal weight) and resin System 1 (Araldite LY 556 + Aradur 917 + DY 070), using vacuum infusion.

Artificial damage was introduced to the specimens using water-jet cutting, creating a circular defect with a diameter of 30 mm at the center. The damaged panels were repaired with circular CFRP patches with a diameter of 45 mm (12 plies G0926, resin System 2 with CSR), also produced using water-jet cutting. Two patch thicknesses were tested to assess their impact on repair efficiency.

- Repaired 1:1: Patch thickness matching the specimen (5 mm);
- Repaired 1:2: Patch thickness half that of the specimen (2.5 mm).

The patches were bonded to the panels using Araldite 2031 (Salt Lake City, UT, USA), a two-component epoxy adhesive. For reference, some panels without damage served as control samples. A summary of the key characteristics of the panels and repair configurations is provided below (Table 4).

### 2.3. Mechanical Testing

The three-point bending test for panel specimens was conducted in accordance with ASTM D790 [18]. Test specimens were fabricated with 8 layers of G0926 fabric and cut to dimensions of 125 mm in length, 12.51 mm in width, and 3.05 mm in thickness. In this setup, the load is applied at the midpoint between two support points. The flexural strength σ_f_ is calculated using the following equation

(2) $$\sigma_{f}(MPa)=\frac{3PL}{2bd^{2}}\;$$

where

- P: Maximum applied load (N);
- L: Support span (mm);
- b: Width of the specimen (mm);
- d: Thickness of the specimen (mm).

The flexural modulus E_f_ is determined as

(3) $$E_{f}(MPa)=\frac{L^{3}m}{4bd^{3}}\;$$

where m (N/mm) is the slope of the initial straight portion of the load–deflection curve.

For the tensile test, specimens were prepared and tested following ASTM D638 (Supplementary file Figure S2). The tensile machine had a maximum load capacity of 100 kN and operated under ambient conditions. Specimens were cut from the panels in a “dog bone” shape to ensure even stress distribution during testing. The dimensions were 170 mm in length, 13 mm in width, and 3 mm in thickness. The tensile strength σ_t_ is calculated using

(4) $$\sigma_{t}(MPa)=\frac{P}{A}\;$$

where

- P: Maximum applied load (N);
- A: Cross-sectional area of the specimen (mm^2^).

The tensile modulus E_f_ is derived from the slope of the stress–strain curve.

For the impact test, specimens followed the ISO 180 standard [19], which specifies methods for determining the Izod impact resistance of materials (Supplementary file Figure S3). The specimens had dimensions of 80 mm in length, 10 mm in width, and 4 mm in thickness, with no notch. This test measures the energy absorbed by the specimen during fracture when struck by a pendulum. During the impact test, four distinct types of fractures can occur in the specimens [19]. These are as follows.

- Complete fracture (C): The specimen is fully broken into two or more separate pieces.
- Incomplete fracture (H): The specimen is partially fractured, with the two sections connected by a thin, flexible layer of low stiffness.
- Partial fracture (P): The specimen exhibits a break where the two sections are held together by a thicker, sturdier layer compared with the incomplete fracture.
- Non-fracture (N): The specimen does not break; instead, it undergoes bending without separation.

The impact strength I_s_ is calculated using

(5) $$I_{s}(kJ/m^{2})=\frac{E}{bd}\;$$

where

- E: Energy absorbed during fracture (J);
- b: Width of the specimen (mm);
- d: Thickness of the specimen (mm).

For the fracture toughness test, specimens were prepared in accordance with ASTM D5528 [20], which evaluates the Mode I interlaminar fracture toughness (G_I_) of composites using the double cantilever beam (DCB) test (Supplementary file Figure S4).

**Figure 2:**
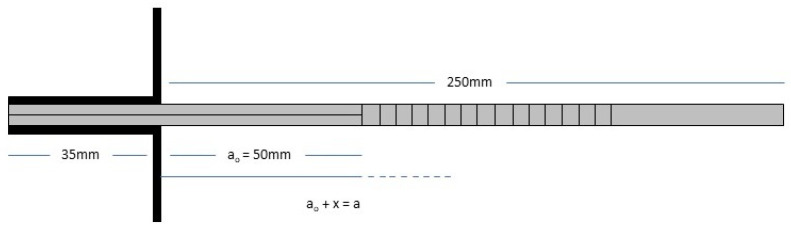
Specimens’ dimensions and geometry for the fracture toughness test.

This test is designed to evaluate composite materials by determining the energy release rate (G_I_) as a function of crack length (α) during crack propagation. The specimens’ dimensions and geometry are shown in Figure 2, with the pre-crack region α_0_ extending to an initial delamination length of 50 mm and a specimen thickness of 4.5 mm. To ensure proper alignment and load application, two piano hinges were bonded to the pre-cracked edges using a two-part toughened epoxy adhesive. The adhesive was cured for 24 h at room temperature.

During testing, the energy absorbed by the specimen (G_I,0_) increases from the point of crack initiation until it reaches a steady-state plateau (G_I,ss_). The relationship between energy and crack length is represented by the R-curve. Crack propagation begins at a critical bridging stress (σ_0_) and develops within a fiber bridging zone described by a non-linear relationship. This continues until the maximum opening displacement (δ_c_) is reached. Beyond this point, the fibers lose their capacity to transfer load and fail, leaving a portion of the crack to propagate without fiber bridging [21].

In fibrous composite materials, the extension of delamination typically follows the fiber bridging mechanisms. For instance, fibers aligned along the crack act to counter its growth by applying opposing forces that attempt to close the crack. This bridging effect influences the extent of crack propagation observed in fracture toughness tests. The R-curve demonstrates this behavior, showing an increase in resistance after crack initiation until a steady-state plateau is reached. However, this curve is dependent on the specimen’s geometry and should not be considered a fundamental property of the material [22].

During testing, thin vertical reference lines, spaced at 5 mm intervals, were marked on the lateral sides of the specimens to aid in crack length measurements. The critical strain energy release rate G_IC_ is calculated using the corrected beam theory as

(6) $$G_{IC}\;(J/m^{2})=\frac{\left(3P\delta \right)}{2b\;(a+|\Delta |)}$$

where

- P: Applied load (N);
- δ: Displacement at the loading point (mm);
- b: Specimen width (mm);
- a: Crack length (mm);
- |Δ|: Correction factor (mm).

The correction factor |Δ| is determined experimentally by constructing a plot of the cube root of compliance (C^1/3^) as a function of the crack length (α). Compliance (C) is calculated as the ratio of the displacement (δ) to the applied load (P). For each crack length, compliance values are determined using the corresponding load and displacement data. The plot of C^1/3^ versus α is then created, and the correction factor |Δ| is obtained as the horizontal intercept of the least-squares linear fit.

The short-beam test was conducted to evaluate the ILSS of the composite specimens according to ASTM D2344 [23] (Supplementary file Figure S5). Composite specimens were fabricated using UD-C415 fabric reinforcement and cut from the composite panels with dimensions of 25 mm in length, 10 mm in width, and 4.5 mm in thickness. A load was applied at a constant crosshead speed of 1 mm/min. The ILSS was calculated using the following formula

(7) $$ILSS\;\left(MPa\right)=\frac{0.75P_{max\;}}{bh}\;$$

where

- P_max_: Maximum load sustained by the specimen (N);
- b: Specimen width (mm);
- h: Specimen thickness (mm).

After testing, the failure modes of the specimens were examined to identify delamination, shear cracking, or other failure mechanisms within the specimen, as indicated by the standard.

Fracture analysis was performed on samples after the flexural, tensile, and short-beam test to determine the fracture mechanism using Scanning Electron Microscopy (SEM) with a tabletop microscope TM3030 (Hitachi, Tokyo, Japan).

The drop-weight impact test (Supplementary file Figure S6) was conducted to evaluate the damage resistance and failure behavior of pristine (reference) and repaired composite panels (Repaired 1:1 and Repaired 1:2). All tests followed the procedure outlined in ASTM D7136 [24], the standard for determining the damage resistance of fiber-reinforced polymer matrix composites subjected to a concentrated impact force. The tests were performed using a drop-tower impact testing machine, FW Magnus 1000 (Coesfeld Material Test, Dortmund, Germany), equipped with a cylindrical impactor weighing 10 kg. During the test, each panel was securely mounted on the fixture to prevent movement during impact, ensuring consistent testing conditions. Initial testing was conducted at varying falling heights to determine the most appropriate energy level for final testing. Preliminary testing revealed that a falling height of 1 m caused excessive damage, making it unsuitable for meaningful comparisons across specimens. Conversely, a height of 0.6 m resulted in insufficient energy to induce measurable damage in some specimens. According to these observations, a height of 0.8 m was selected as optimal for assessing the damage tolerance and energy absorption of the panels. This height delivered an initial impact energy (E) of approximately 78.48 J, calculated using the following formula

(8) $$E\;\left(J\right)=mgh\;$$

where

- m: Mass of the impactor (kg) = 10 kg;
- g: Gravitational acceleration (m/s^2^) = 9.81 m/s^2^;
- h: Falling height (m) = 0.8 m.

Throughout the test, data were collected using the system’s sensors, which recorded load, displacement, and energy metrics throughout the impact event. After each test, all panels were visually inspected to document damage characteristics. Observations included delamination, matrix cracking, fiber breakage, and penetration, with both the front and back surfaces of the panels carefully examined to ensure detailed damage evaluation.

### 2.4. Non-Destructive Testing

After visually inspection of the impacted composite panels described in Section 2.3, the same specimens were subjected to NDT using ultrasonic inspection. This method allows for the detection of internal defects, such as cracks, delamination, and other flaws, without further damaging the specimens. NDT was conducted using a Dolphicam2 portable ultrasonic C-scanner (Dolphitech, Gjøvik, Norway), coupled with its proprietary software. The technical specifications of the Dolphicam2 system are provided in Table 5. The transducer module that was used was 5 MHz. The suitable gain for this visualization was found to be 1 dB, and the obtained images were based on the time-of-flight value. Finally, as a contact agent, an ultrasonic couplant gel was chosen.

## 3. Results

### 3.1. Flexural Test

The three-point bending test was conducted on five specimens per type. The key results are summarized in Table 6.

**Figure 3:**
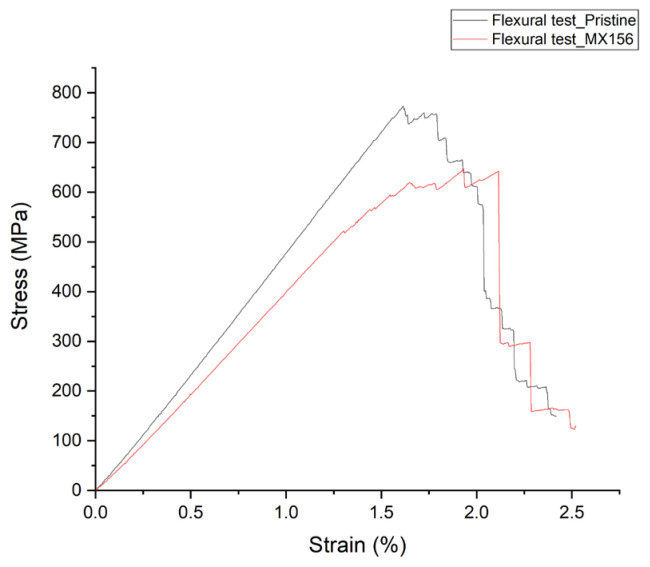
Indicative stress–strain curve from the three-point test.

The stress–strain response for each type of specimen during the bending test is illustrated in Figure 3.

The incorporation of core–shell elastomers in the MX156 resin system resulted in a noticeable reduction in both the flexural strength and modulus of elasticity of the composites when compared with the reference specimens made with pristine resin. Specifically, a 13.8% decrease in flexural strength was observed, which is attributed to the presence of elastomeric particles. These particles introduce localized discontinuities within the epoxy matrix, compromising its load-bearing capacity and, consequently, that of the composite as a whole. Similarly, the reduction in the elastic modulus indicates a softening effect induced by the elastomers, as they alter the stiffness of the resin matrix [11].

Despite the observed reductions, the strain at failure for the MX156 specimens was higher than that of the pristine specimens, increasing by approximately 26%. This suggests that the addition of core-shell particles enhances the ductility of the composite, allowing it to sustain greater deformation before failure. The trade-off between reduced strength and improved strain at failure is consistent with the toughening mechanisms introduced by the elastomeric inclusions [25].

The observed decrease in flexural strength and stiffness should be considered when designing CFRP components for structural applications. While the MX156 system may not be ideal for applications where stiffness and strength are critical, the increase in strain at failure offers significant advantages in applications requiring ductility and damage tolerance, such as components subject to cyclic loading or localized bending stresses. The long-term impact of reduced stiffness may also depend on the operational environment; for instance, the MX156 resin system could offer improved resistance to sudden impacts or dynamic loads due to its enhanced energy-dissipating properties.

Overall, the small reduction in strength and modulus indicates that the type of carbon fiber used has a strong affinity with the resin systems, ensuring good load transfer between the matrix and reinforcement. This compatibility minimizes the negative effects of resin modifications, preserving the overall performance of the composite material.

### 3.2. Tensile Test

As in the three-point bending test, tensile tests were performed on pristine and MX156 composite specimens, with the results summarized in Table 7.

**Figure 4:**
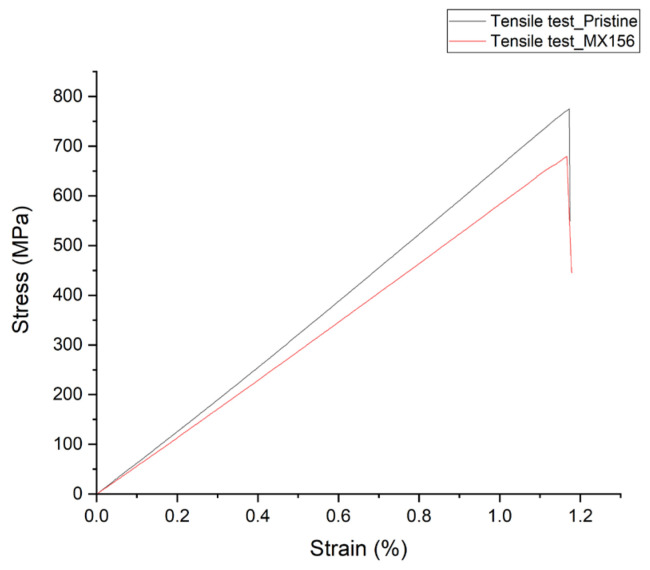
Indicative stress–strain curve from tensile tests.

The stress–strain curves in Figure 4 reveal distinct differences between the mechanical behavior of the pristine and MX156 specimens. Composites with MX156 resin exhibited a 12.5% reduction in maximum tensile strength compared with their pristine counterparts. This decrease in strength is attributed to the inclusion of core–shell particles within the resin matrix, which introduce localized discontinuities that disrupt stress transfer across the composite. Similar to the behavior observed in the bending test, CSR acts as weak points under tensile loading, reducing the overall capacity of the material to bear a load. This decrease in tensile strength may limit the use of MX156 in applications where the primary loading mechanism involves direct tensile stresses. However, in applications where tensile loads are secondary or where enhanced energy absorption is prioritized—such as crash structures, protective panels, or components exposed to dynamic stresses—the MX156 system could provide significant advantages.

In addition to the reduction in tensile strength, the Young’s modulus of the MX156 specimens was also slightly lower than that of the pristine specimens, decreasing by approximately 7.1%. This indicates a softening effect introduced by the elastomeric core–shell particles, which slightly reduced the stiffness of the matrix and, consequently, the composite. Despite this, the strain at failure remained nearly identical between the two samples.

The strain behavior observed in the tensile test contrasts with that of the flexural test, where the strain at failure increased for MX156 specimens. This difference arises from the distinct loading mechanisms in these two tests. In the flexural test, the matrix plays a larger role in resisting deformation, particularly on the tension side of the specimen. The core–shell particles enhance the resin’s ability to accommodate deformation locally, resulting in increased strain at failure. In tensile loading, however, the CF reinforcement dominates the mechanical response, with the resin primarily transferring stress between fibers. The core–shell particles have less influence on the overall tensile ductility, as the load-bearing capacity is governed predominantly by fiber reinforcement. The relatively small reduction in the tensile properties, despite the matrix modifications, highlights the dominant role of CF reinforcement in determining the overall tensile behavior of the composite. In CFRPs, the fibers bear the majority of the applied load, with the matrix primarily serving to bind the fibers together and transfer stress between them [26].

Glaskova-Kuzmina et al. [10] investigated the addition of CSR (MX156) to pure epoxy at concentrations of 2%, 4%, and 6%, observing a reduction in tensile strength ranging from 10% to 20%, depending on the CSR concentration. Their results also revealed a significant increase in strain at failure from 4.9% to a maximum of 7.2%, showcasing the ductility-enhancing effect of the CSR particles in epoxy systems. On the other hand, Hyeongcheol Park et al. [27] studied the incorporation of CSR into CFRPs at concentrations up to 20%, reporting a decrease in tensile strength of 6% to 20%. Their findings align with this work, confirming that the reinforcing effect of carbon fiber is the dominant factor in determining the tensile properties of toughened CFRPs.

### 3.3. Impact Test (Pendulum)

The results of the impact test for both types of specimens are summarized in Table 8.

The values represent the average of the samples tested, all of which exhibited partial fractures. The specimens with MX156 resin demonstrated a significant improvement in impact strength, showing a 49.8% increase compared with those with pristine resin. This enhancement is attributed to the effectiveness of the CSR particles in reinforcing the epoxy matrix under impact loading conditions. Similarly, in the study by Hyeongcheol Park et al. [27], impact tests on CFRPs containing CSR at concentrations up to 20% showed an even greater improvement in impact strength, with increases reaching up to 87.5%.

The improved impact resistance of the MX156 specimens can be attributed to the unique structural and energy-dissipating properties of the CSR particles. Epoxy resins, known for their highly cross-linked molecular structure, typically exhibit low fracture toughness due to their inability to resist crack propagation effectively. This limitation often reduces the strength of composites under impact loading. However, the inclusion of CSR particles in the resin mitigates this issue through several toughening mechanisms.

Firstly, the elastomeric nature of the CSR particles allows them to absorb significant amounts of energy and deform under stress, thereby enhancing the composite’s overall ability to resist impact. Additionally, the hollow core of the CSR particles contributes to energy absorption during impact, as the cavity can dissipate further energy through localized deformation. Lastly, the well-dispersed CSR particles act as crack terminators within the epoxy matrix. These particles effectively terminate crack growth during mechanical stress, preventing further propagation and maintaining the structural integrity of the composite [27,28].

Despite exhibiting the same type of partial breakage as the pristine resin composites, the MX156 specimens demonstrated superior impact resistance due to the toughening mechanisms provided by the CSR particles. This makes the MX156 resin system a valuable choice for applications requiring enhanced energy absorption and impact performance in composite materials.

In practical applications, enhanced impact resistance makes the MX156 resin system particularly suitable for CFRPs used in industries where materials are exposed to sudden or repeated impact loads. Examples include the automotive and aerospace sectors, where crashworthiness and energy absorption are critical, as well as protective panels in wind turbine blades or sports equipment.

### 3.4. Fracture Toughness Test

**Figure 5:**
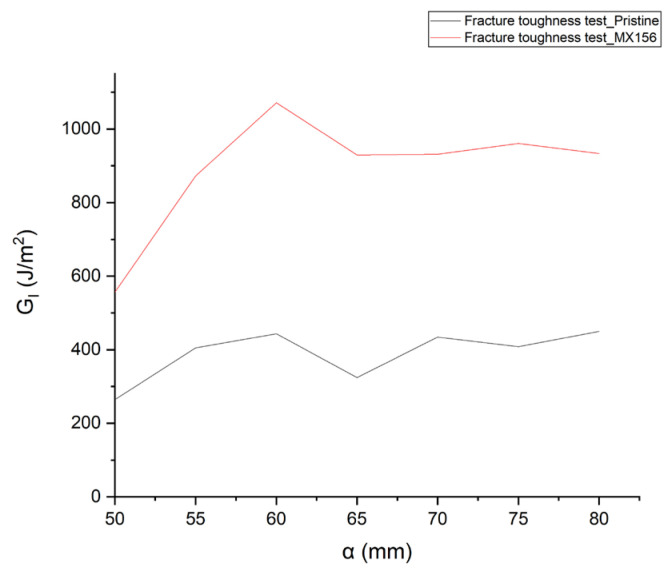
Indicative GI–α graph for pristine and MX156 samples.

The G_I_–α table (Table 9) was constructed after calculating the energy release rates for specific crack lengths (α), starting from 50 mm and increasing in increments of 5 mm. The values and corresponding graph (Table 9, Figure 5) for the two types of specimens—those with pristine resin and those with MX156 resin containing CSR particles—are presented, based on the corrected beam theory.

The energy absorbed by the specimens during the Mode I test increases dramatically in the presence of CSR elastomers. This behavior arises because the CSR-modified resin alters the mechanisms involved in the fracture process of the composite material. Comparing the energy release rates of the two types of specimens, it is evident that the MX156 resin specimens absorb up to 181% more energy during crack propagation compared with those with the pristine resin. This behavior arises because the CSR-modified resin alters the mechanisms involved in the fracture process of the composite material. This substantial increase is attributed to the ability of CSR particles to act as energy absorbers.

The core-shell particles contain elastic-phase cores with cavities that enhance energy dissipation during crack propagation. These cavities are responsible for the fluctuations observed in the G_I_ values of the MX156 specimens, as opposed to the relatively stable energy release behavior observed in pristine specimens. In the latter case, the resistance to crack propagation is primarily provided by the fiber bridging zone, which is the dominant mechanism in fiber–matrix systems without CSR modifications [29].

The core–shell particles not only absorb impact energy but also act as crack terminators by inhibiting crack propagation through mechanical dissipation. This tendency is evident in the fluctuations observed in the corresponding R-curves, where the energy absorbed compensates for crack propagation resistance. The MX156-modified composites exhibit significantly higher energy absorption peaks during crack growth due to the active role of the CSR cavities in mitigating crack propagation.

From the graphs and data, it can be concluded that while the shape of the G_I_–α curve remains consistent, irrespective of the method used to calculate G_I_, the absolute values of G_I_ differ substantially between the specimens. The CSR-modified MX156 resin demonstrates a consistent and pronounced improvement in fracture toughness, with the ability to absorb more than double the energy compared with pristine resin.

The study by Glaskova-Kuzmina et al. [10] demonstrated that among CFRPs with woven fabric incorporating 2%, 4%, and 6% CSR, the optimal performance was achieved with 4% CSR, yielding a 32% improvement in impact resistance. Similarly, Pascal Van Velthem et al. [30] reported a 25% increase in impact resistance using CFRPs with 10% CSR and woven fabric. In comparison, the 25% CSR content used in this work resulted in a significantly higher improvement in impact resistance, mainly due to the higher concentration of CSR, but this can also be partly attributed to the uniform dispersion of CSR particles achieved through a well-optimized infusion process. In contrast, the study by Van Velthem et al. observed a high concentration of CSR in the upper layers of the composite due to an inefficient infusion technique, which likely limited the overall enhancement in mechanical performance [11].

### 3.5. Short-Beam Test

The short-beam test was conducted to determine the ILSS of composites that failed by interlaminar shear, and the results are summarized in Table 10.

The ILSS decreases slightly in the presence of CSR nanoparticles compared with the specimens containing the unmodified three-component resin. On average, the MX156 specimens exhibit 9.9% lower ILSS compared with the pristine specimens.

**Figure 6:**
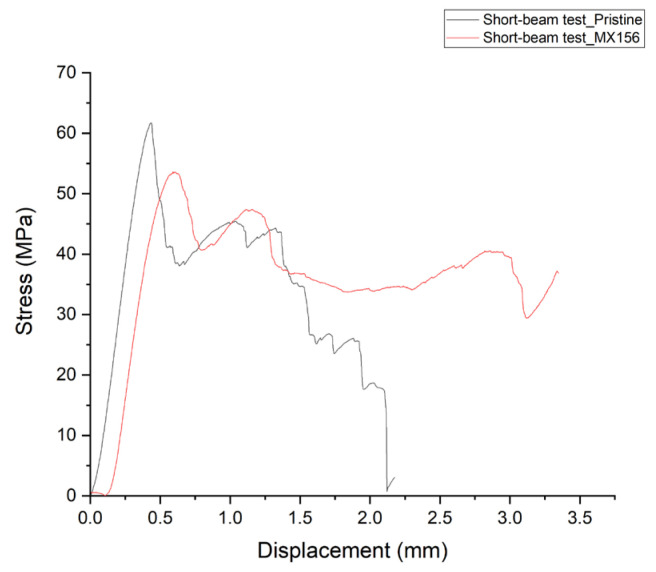
Indicative stress–displacement curve of pristine and MX156 samples.

Despite the slightly lower ILSS of the MX156 samples compared with the pristine ones, an important observation is that, after a certain displacement value, the shear strength in the MX156 specimens stabilizes, forming a plateau (Figure 6). This plateau behavior is absent in the pristine specimens, where the shear strength shows more pronounced drops after reaching the peak load. This difference can be attributed to the unique properties of the CSR nanoparticles in the MX156-modified composites. These nanoparticles act as crack terminators, inhibiting the propagation of cracks that develop within the composite material. Consequently, they contribute to extending the lifespan of the composite by stabilizing its mechanical performance under shear loading. In contrast, the pristine resin specimens rely solely on the interaction between the epoxy matrix and the fibers to resist shear forces, resulting in less consistent performance after the peak load.

### 3.6. Fracture Analysis

**Figure 7:**
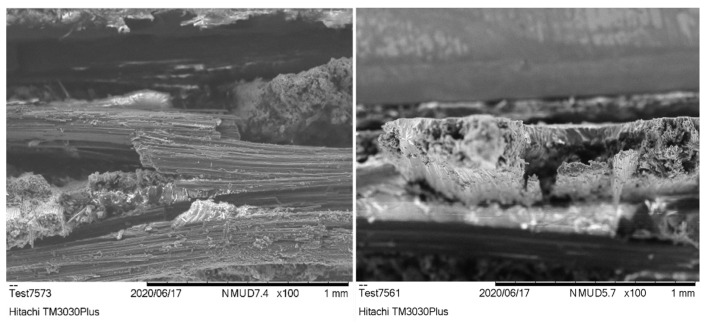
SEM image after the tensile test for (**left**) the pristine sample and (**right**) the MX156 sample.

The fracture surfaces from the tensile test show similar characteristics for both the pristine and MX156 samples (Figure 7). In both cases, the fibers appear cleanly broken, with visible matrix residue on their surfaces. This suggests that both resin systems provide adequate fiber–matrix adhesion, with no significant differences in terms of exposed matrix or wetting ability. The failure appears to be dominated by fiber breakage, as expected for tensile loading where the stress is largely carried by the fibers. The presence of CSR particles in the MX156 specimens does not seem to alter the failure mechanism compared with the pristine specimens. The similar fracture patterns observed are consistent with the mechanical test results, where the tensile strain at failure remained nearly identical between the two resin systems. This suggests that the reinforcement dominates the tensile behavior, with the matrix primarily functioning as a binder to transfer stress between the fibers.

**Figure 8:**
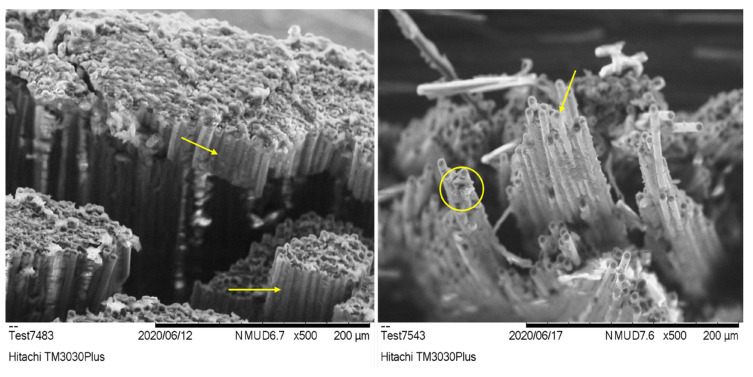
SEM image after the flexural test for (**left**) the pristine sample and (**right**) the MX156 sample.

The SEM images of the fracture surfaces from the flexural test of both sample types exhibit good resin coverage on the exposed fibers, indicating good wetting and impregnation of the fibers by the resin (Figure 8). This consistency between the two resin systems confirms that the CF reinforcement interacts effectively with both matrices, ensuring efficient load transfer during flexural loading.

The fracture surface of the pristine specimens is dominated by brittle failure, characterized by sharp matrix cracking and fiber pull-out. The clean separation of the fibers suggests that the matrix failed abruptly (as indicated by the yellow arrows in Figure 8 left), transferring the load directly to the fibers, which then fractured due to tensile overstress on the tension side of the specimen. This brittle behavior aligns with the higher flexural strength but lower strain at failure observed in the mechanical tests, as the matrix lacks the ductility to absorb significant deformation before failure.

In the MX156 specimens, the fracture surface shows similar failure mechanisms, with no notable improvement in fiber–matrix adhesion compared with the pristine samples. However, the presence of core–shell elastomers in the MX156 matrix contributes to greater matrix deformation around the fibers, visible as more resin distortion in the fracture plane (as indicated with yellow circle and arrow in Figure 8 right). This increased deformation reflects the toughening effect of the CSR particles, which allow the matrix to absorb more energy before failure. Despite this, the overall flexural strength is reduced due to the softening effect introduced by the elastomers, which is consistent with the mechanical test results.

**Figure 9:**
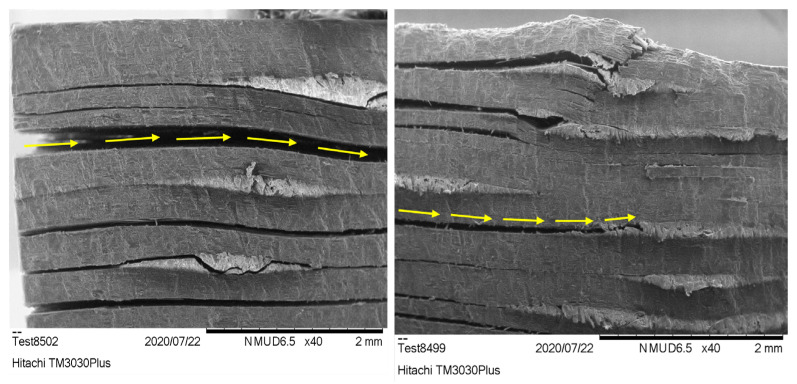
SEM image after the short-beam test for (**left**) the pristine sample and (**right**) the MX156 sample.

The SEM images from the short-beam test reveal notable differences between the pristine and MX156 specimens (Figure 9). In the pristine specimens, interlaminar cracking and delamination are prominent, with distinct separation between layers, indicating limited interlaminar shear resistance (as highlighted with yellow arrows in Figure 9 left). In contrast, the MX156 specimens show less delamination and a smoother transition between layers, reflecting improved toughness (as highlighted with yellow arrows in Figure 9 right). This behavior aligns with the plateau observed in the ILSS mechanical results, where the crack propagation stabilizes in the MX156 specimens, further delaying catastrophic failure. The CSR particles contribute to crack termination and energy absorption, enhancing the matrix’s ability to resist interlaminar shear forces, even though the ultimate ILSS value is slightly lower compared with the pristine system.

### 3.7. Drop-Weight Impact Test

As described in Section 2.3, the theoretical energy calculated for the impact test was 78.5 J, but the experimental results indicate lower energy absorption in all cases. This discrepancy can be attributed to energy losses during the test. Friction in the testing system, energy dissipation as sound waves, and heat generated during impact contribute to the reduction in measured energy. Additionally, the energy absorbed varies between the cases due to the mechanical properties and structural configurations of the samples.

When comparing the peak loads across the specimens (Table 11), the reference (F_m_ = 18.17 ± 1.86 kN) and Repaired 1:1 (F_m_ = 17.58 ± 0.79 kN) samples exhibit nearly identical values, indicating that the repair patch used in Repaired 1:1 specimens effectively restores the load resistance of the damaged panels. This also suggests that the CSR-modified resin in the patch maintains the structural integrity of the repaired panel under impact loads. On the other hand, the Repaired 1:2 specimens demonstrate significantly lower peak loads (F_m_ = 8.68 ± 0.46 kN), highlighting the inadequacy of the thinner patch in resisting impact forces.

In terms of energy absorption, the Repaired 1:1 samples (E_m_ = 59.62 ± 3.63 J) outperform the reference specimens (E_m_ = 43.42 ± 6.54 J) by approximately 37%, reflecting the enhanced toughness imparted by the CSR-modified resin. The plateau-like behavior observed in the load–time and load–displacement graphs supports this improvement, indicating sustained resistance to crack propagation. Conversely, the Repaired 1:2 specimens absorb the least energy (E_m_ = 23.90 ± 2.69 J), capturing only 30% of the theoretical energy. This poor performance is due to the insufficient thickness of the patch, which cannot effectively distribute or absorb the impact energy, leading to premature failure.

**Figure 10:**
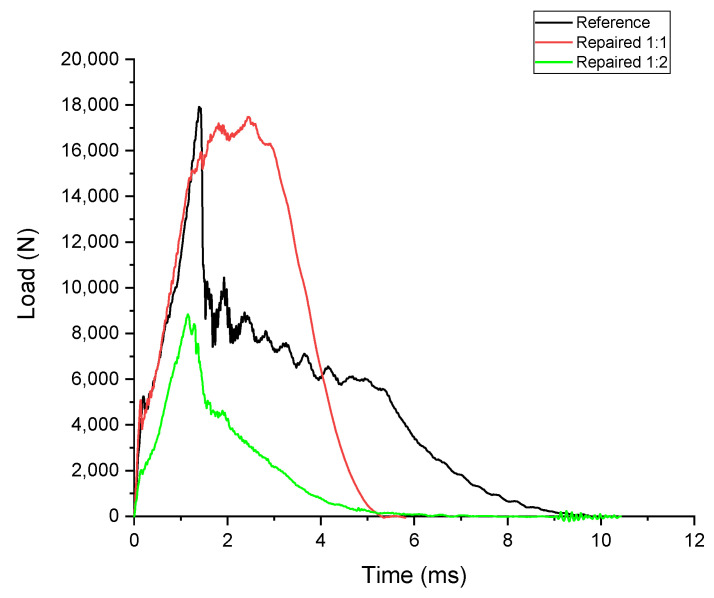
Indicative drop-weight impact test results on the reference (black), Repaired 1:1 (red), and Repaired 1:2 (green) panels: Load vs. time.

The load–time graph (Figure 10) reveals that both the reference and Repaired 1:1 specimens reach their peak loads quickly and maintain a sharp initial increase. However, while the reference specimens exhibit a steep drop immediately after the peak, indicating brittle failure, the Repaired 1:1 samples show a plateau following the peak load. This behavior reflects the ability of the CSR-modified resin in the patch to delay crack propagation and sustain the load for a longer duration. In contrast, the Repaired 1:2 specimens display a much lower peak load and a rapid decline after the peak, confirming their inability to resist or dissipate impact energy effectively.

**Figure 11:**
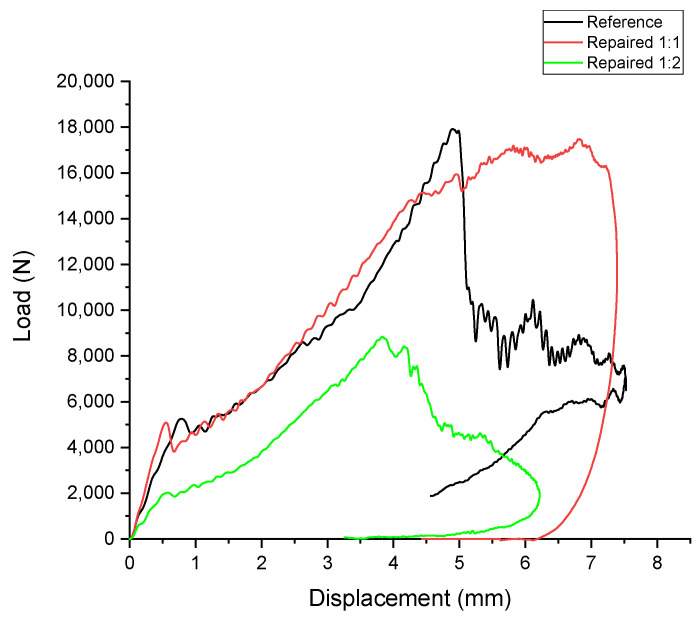
Indicative drop-weight impact test results on the reference (black), Repaired 1:1 (red), and Repaired 1:2 (green) panels: Load vs. displacement.

Similar to the load–time graph, for the load–displacement graph (Figure 11), both the reference and Repaired 1:1 specimens exhibit a steep initial increase in load, reflecting their high stiffness. The Repaired 1:1 samples demonstrate a plateau after the peak, indicating greater ductility and sustained energy absorption due to the presence of CSR particles in the resin. However, a notable difference is observed in the return slopes after failure. For the reference specimens, which are penetrated (four out of five specimens), the return slope is steep, indicating minimal plastic deformation and rapid unloading, as shown by the sharp decrease in displacement values. In contrast, the Repaired 1:1 specimens, which are not penetrated, show a gentler return slope. This behavior suggests greater plastic deformation, consistent with the enhanced toughness of the CSR-modified resin, which allows the material to absorb more energy and recover more gradually. The Repaired 1:2 specimens, however, exhibit a shallow initial slope, rapid failure, and a steep return, further confirming their inability to sustain loads or dissipate energy effectively.

**Figure 12:**
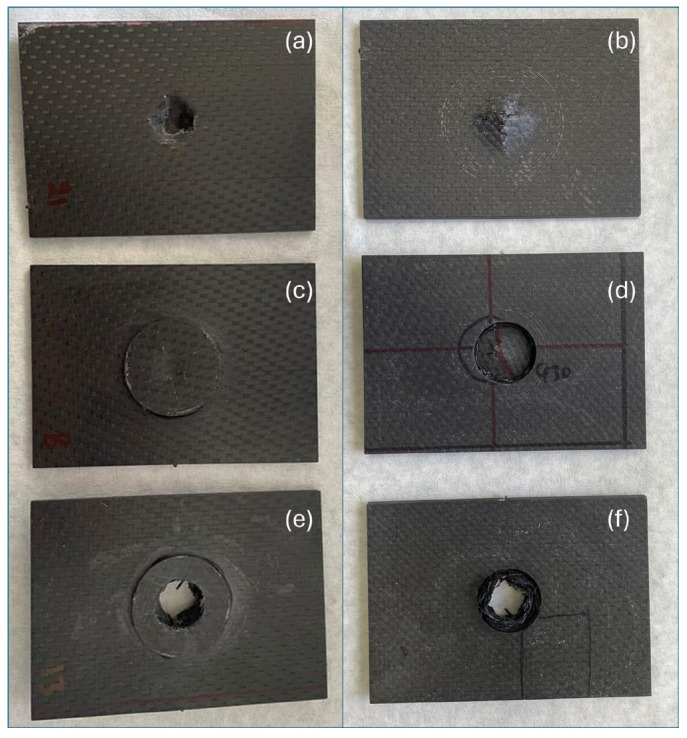
Indicative impacted co mposite specimens: front view of (**a**) the reference, (**c**) Repaired 1:1, and (**e**) Repaired 1:2 samples, and back view of (**b**) the reference, (**d**) Repaired 1:1, and (**f**) Repaired 1:2 samples.

The visual inspection further validates these findings (Figure 12). The Repaired 1:1 specimens displayed only localized indentation, with no penetration observed on any of the panels. This indicates that the thicker CSR-modified patch effectively absorbed and dissipated the impact energy, preventing catastrophic failure. The damage was contained within a relatively small area with a circular and shallow indentation pattern, suggesting controlled energy transfer across the composite surface. In contrast, the Repaired 1:2 specimens exhibited complete penetration in all panels, with larger and irregular perforation shapes. This confirms that the thinner patch lacks the structural integrity required to resist the applied impact energy. The damage in these specimens extends radially outward from the impact site, reflecting lower energy dissipation and reduced resistance to crack propagation. For the reference panels, four out of five samples were penetrated, while one showed damage resembling indentation. The penetration in the reference panels is characterized by a more irregular and sharp-edged break compared with the smoother penetration observed in the Repaired 1:2 specimens. This suggests that the lack of CSR particles in the matrix of the reference panels results in brittle failure, with less ability to dissipate energy effectively. The indentation observed in one panel aligns with the overall lower impact strength and variability in behavior observed for the reference specimens.

### 3.8. Ultrasound

**Figure 13:**
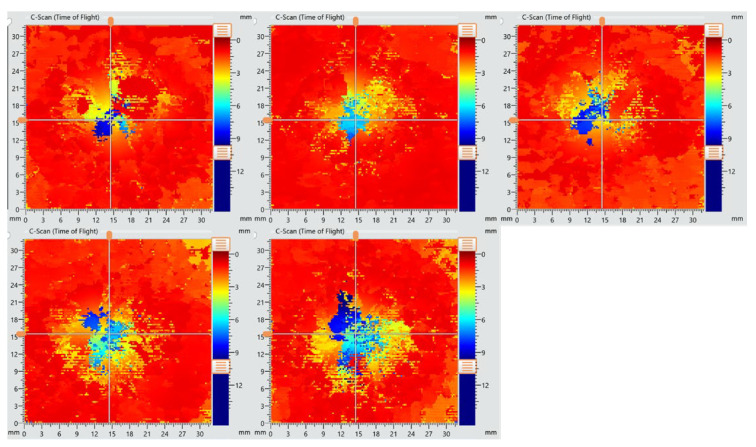
C-scan images of reference specimens.

**Figure 14:**
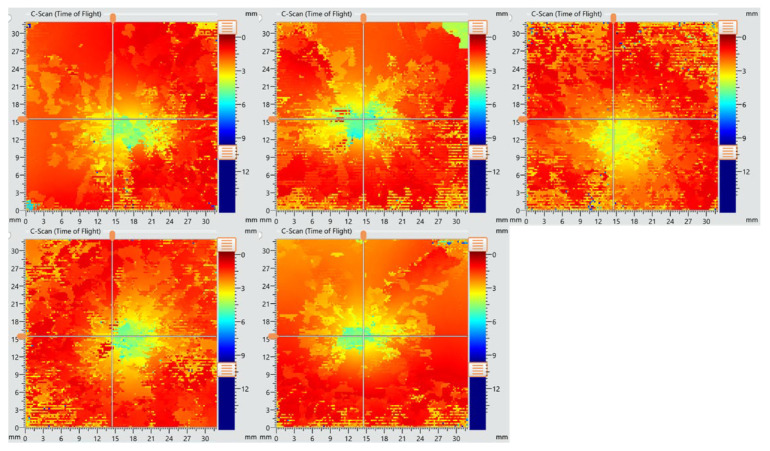
C-scan images of Repaired 1:1 specimens.

The objective of ultrasonic testing was to examine the extent of damage caused by the impact and to assess how it propagated through the surrounding material of the impact site. The results focus on interlaminar delaminations and their depth distribution within the composite panels. The C-scan images of the NDT conducted for this purpose are presented in Figure 13 for the reference samples and Figure 14 for the Repaired 1:1 samples. The scale on the right of each image indicates the depth of the delaminations: orange corresponds to damage closest to the transducer probe (near the surface), while dark blue represents defects located deeper within the panel, closer to the back side.

The C-scan images for the reference specimens (Figure 13) reveal significant damage spreading across a large area surrounding the impact site. The C-scan images for the reference specimens (Figure 13) reveal significant damage spreading across a large area surrounding the impact site, with evidence of deep penetration into the thickness. This is confirmed by the presence of dark blue zones, which indicate delaminations near the back side of the panel.

In contrast, the Repaired 1:1 specimens (Figure 14) show a significantly reduced extent of delamination and damage. The area of delamination is confined to roughly 30% of the scanned impacted area, considerably smaller than that of the reference samples. Additionally, the penetration depth is less severe, with zero dark blue regions. Most delaminations are localized closer to the front and middle layers, as indicated by green and yellow in the images. This reduced extent and depth of damage can be attributed to the CSR-modified resin in the repair patch, which effectively dissipates energy and limits crack propagation. The damage morphology observed in the Repaired 1:1 samples correlates well with the indentation-like failure seen in the visual inspection, confirming the effectiveness of the thicker patch in absorbing impact forces and preventing catastrophic failure.

## 4. Conclusions

This study emphasizes the dual potential of CSR-modified matrices for improving the intrinsic properties of CFRPs and enabling efficient repair solutions for damaged composites. The incorporation of CSR into the epoxy matrix of CFRPs significantly enhances their impact resistance and fracture toughness, by approximately 50% and up to 181%, respectively, offering an effective method for improving the durability of composite materials. While slight reductions of 13.8% and 12.5% in flexural and tensile strengths were observed due to the softening effect of the CSR-modified matrix, these compromises are offset by considerable gains in ductility, energy absorption, and damage tolerance.

The enhanced impact resistance of MX156-based CFRPs makes them particularly well-suited for applications requiring damage tolerance and durability, such as aircraft structures, automotive body panels, and energy-absorbing crash components. However, the observed decreases in flexural and tensile strengths indicate that this resin system may not be the best choice for applications requiring high stiffness or strength under static loading. Instead, the MX156 system should be targeted for use in dynamic or impact-critical applications where its energy-dissipating properties and improved toughness provide a significant advantage.

Patch repair was demonstrated to be an effective technique for restoring the structural integrity of damaged CFRPs. Repairs with CSR-modified patches of equal thickness to the panel not only restored the load-bearing capacity but also improved energy absorption by 37.3% compared with the reference samples. Moreover, C-scans of these samples revealed reduced delamination and damage depth. The thinner patches, however, were inadequate for sustaining impact loads, as confirmed by both mechanical testing and ultrasonic C-scan analysis.

A key area for future exploration is the long-term durability of CSR-modified CFRPs when exposed to environmental conditions such as temperature fluctuations and humidity, which are critical for their real-world application. Additionally, optimizing patch repair techniques, including exploring alternative adhesives or geometries, could further enhance repair effectiveness. Lastly, investigating the recyclability of CSR-modified composites and their impact on the recycling process would contribute to the sustainable development of CFRPs, addressing both performance and environmental concerns.

## Figures and Tables

**Table 1:** Characteristics of fabrics.

Code	Type of Fabric	Yarns	Weave	Weight Distribution (Warp–Weft%)	Nominal Weight (g/m^2^)	Density (g/cm^3^)
G0926	Multiaxial	Warp: CF, TENAX E HTA40 E13 6K Weft: CF, TENAX E HTA40 E13 6K	5H Satin	50–50	375	1.76
C415	Unidirectional	Warp: 12K CF Weft: GF	UD, woven	92–8	415	1.82

**Table 2:** Composition of resin systems.

Resin Systems	Component A (Resin)	Component B (Hardener)	Component C (Accelerator)	Ratio (*w*/*w*%)	Curing/ Post-Curing Conditions
System 1	Araldite LY 556	Aradur 917	DY 070	100:90:0.5	4 h at 80 °C/ 4 h at 120 °C
System 2	Kaneace MX156 (75% resin/25% CSR)			133:90:0.5	4 h at 80 °C/ 4 h at 120 °C

**Table 3:** Characteristics of manufactured CFRPs and fiber volume fraction.

A/A	Matrix	Matrix Mixing Ratio	Fabric Type	Average Thickness (mm)	Nο. of Plies	Nominal Weight (g/m^2^)	Density (g/cm^3^)	Vf (%)	Mechanical Tests
Pristine_ Panel_ 1	Araldite LY 556 + Aradur 917 + Accelerator DY 070	100:90:0.5	G0926	3.05	8	375	1.76	55.89	3-point bend tensile
Pristine_ Panel_ 2			UD C415	4.5	12	415	1.82	60.8	MODE I short beam
Pristine_ Panel_ 3			UD C415	4.5	10	415	1.82	56.5	Impact
MX156_ Panel_ 1	MX156 + Aradur 917 + Accelerator DY 070	133:90:0.5	G0926	3.05	8	375	1.76	58.7	3-point bend tensile
MX156_ Panel_ 2			UD C415	4.5	12	415	1.82	61.19	MODE I short beam
MX156_ Panel_ 3			UD C415	4.5	10	415	1.82	57	Impact

**Table 4:** Features of CFRP panels to be examined.

	Reference	Repaired 1:1	Repaired 1:2
Number of samples	6	7	8
Composite resin system	Araldite LY 556/Aradur 917/DY 070 (100:90:0.5)		
Patch resin system	MX156 + Aradur 917 + Accelerator DY 070 (133:90:0.5)		
Composite/patch reinforcement	G0926		
Composite dimensions, mm (L × W × t)	100 × 150 × 5		
Damage dimensions, mm (D × t)	N/A	30 × 5	30 × 5
Repair patch dimensions, mm (D × t)	N/A	45 × 5	45 × 2.5

**Table 5:** Technical datasheet of the Dolphicam2 system.

Technical Details	
Transducer type matrix	2D-array
Transducer elements	128 × 128 (16.384)
Transducer aperture	32 × 32 mm
Element pitch	250 μm
Center frequency	5 MHz–6 dB
Frequency bandwidth	120%
Sample rate	50 MHz
Acquisition rate A-scans	100.000–500.000 datasets per second
Acquisition rate 3D	10–40 3D volumes per second

**Table 6:** Three-point test results.

Specimen Type	Flexural Strength (MPa)	Modulus (GPa)	Strain (%)
Pristine	768 ± 19	49.2 ± 1	1.84 ± 0.06
MX 156	662 ± 10	39.1 ± 1.6	2.32 ± 0.16

**Table 7:** Tensile test results.

Specimen Type	Tensile Strength (MPa)	Young’s Modulus (GPa)	Strain (%)
Pristine	776 ± 11	61.8 ± 1.3	1.17
MX 156	679 ± 16	57.4 ± 0.6	1.16

**Table 8:** Impact test results.

Specimen Type	Type of Breakage	Impact Strength (kJ/m^2^)
Pristine	P	83.7 ± 12.9
MX156	P	125.4 ± 29.3

**Table 9:** Absorbed energy for pristine and MX156 samples calculated by the CBT.

α (mm)	Pristine G_I_ (J/m^2^)	MX156 G_I_ (J/m^2^)	% Increase Compared with Pristine
50	210 ± 15	590 ± 41	+181
55	310 ± 22	790 ± 55	+155
60	405 ± 28	990 ± 69	+144
65	385 ± 27	890 ± 62	+131
70	415 ± 29	840 ± 59	+102
75	425 ± 30	815 ± 57	+91
80	405 ± 28	795 ± 56	+96

**Table 10:** ILSS of pristine and MX156 samples.

Specimen Type	ILLS (MPa)
Pristine	59.6 ± 4.2
MX156	53.7 ± 1.9

**Table 11:** Peak load and maximum energy absorbed of the drop-weight impact test.

	F_m_ (kN)	E_m_ (J)
Reference	18.17 ± 1.86	43.42 ± 6.54
Repaired 1:1	17.58 ± 0.79	59.62 ± 3.63
Repaired 1:2	8.68 ± 0.46	23.90 ± 2.69

## Data Availability

Data are contained within the article and Supplementary Materials. Further inquiries can be directed to the corresponding author.

## Supplementary Materials

The following supporting information can be downloaded at https://www.mdpi.com/article/10.3390/polym17030407/s1. Figure S1: Composite laminate manufacturing using vacuum infusion; Figure S2: Experimental set-up of Tensile tests; Figure S3: Experimental set-up of Impact tests; Figure S4: Experimental set-up of MODE I tests; Figure S5: Experimental set-up of short-beam tests; Figure S6: Experimental set-up of drop tower tests as performed in Institute of Lightweight Engineering and Polymer Technology, TUD Dresden University of Technology.
