# Supplementary material for: Enhancing Carbon Fiber-Reinforced Polymers’ Performance and Reparability Through Core–Shell Rubber Modification and Patch Repair Techniques
Source: Polymers (Basel). 2025 Feb 3;17(3):407. doi: 10.3390/polym17030407 (PMC11820729; doi:10.3390/polym17030407)
Supplement: Supplementary file 1 [file polymers-17-00407-s001.zip › polymers-3446454-supplementary.pdf]

1. Composite production

The composite laminates were manufactured using the vacuum infusion technique, as illustrated in Figure S1. This process involves spreading the resin uniformly across all layers of carbon fibre fabrics by creating a vacuum within the system. The molding process was carried out on a rectangular mold.

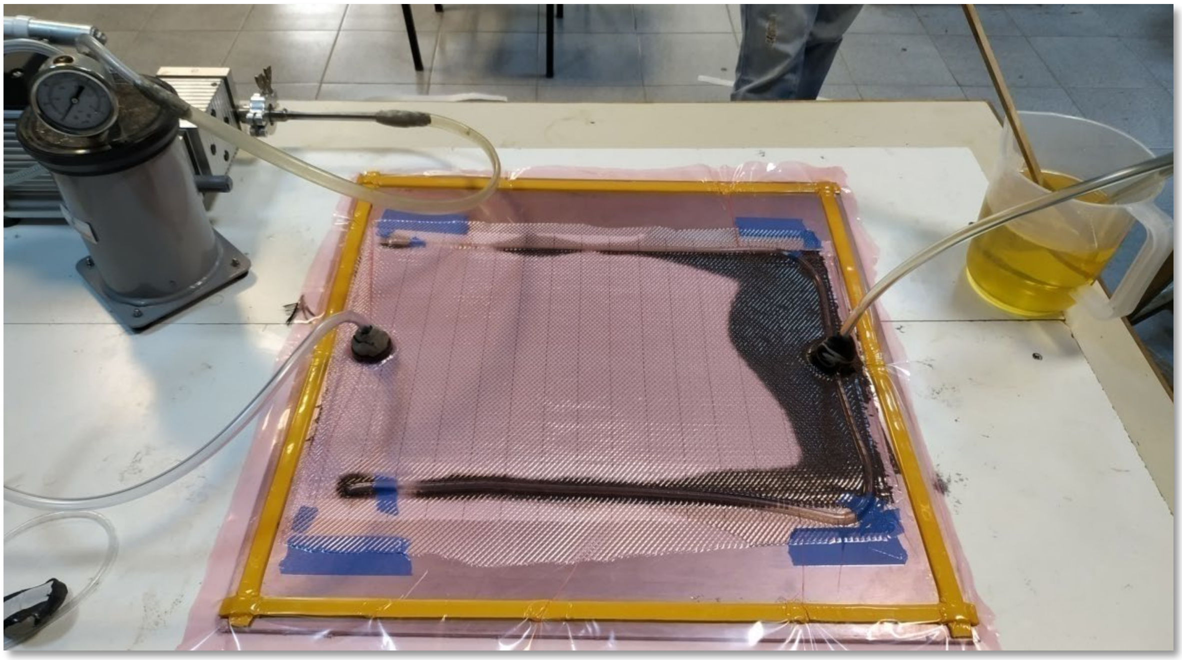

Figure S1 Composite laminate manufacturing using vacuum infusion

Preparation of the Mold and Setup: The procedure began with thorough waxing of the mold using a high-temperature release wax to facilitate easy removal of the composite after curing. Carbon fibre fabrics were stacked layer by layer onto the mold in a dry state, without the resin being added at this stage. On top of the fabric layers, a peel ply was placed to ensure the successful separation of the laminate from the additional consumable layers. To facilitate the uniform flow of resin during infusion, an infusion mesh was added above the peel ply. Finally, the mold was sealed with a vacuum bag, which enclosed the entire setup to prevent air from entering the system during the infusion process.

Vacuum Creation and Resin Infusion: Two openings were made in the vacuum bag. One was connected to a resin trap, which in turn was connected to the vacuum pump. The resin trap served to protect the pump from any resin that might escape during the infusion process. The second opening was connected to a beaker containing the resin to be infused, with all connections made using plastic tubing. Once the setup was complete, air was evacuated from the mold by closing the resin inlet and activating the vacuum pump. The vacuum conditions were monitored using a manometer attached to the resin trap. After achieving vacuum, the resin inlet was opened, allowing the vacuum pump to draw the resin from the beaker into the mold. The resin flowed through the carbon fibre layers and eventually exited into the resin trap, ensuring complete wetting of the fibres.

Curing Process: After the infusion was complete, the mold was placed in an oven for curing. The curing process was conducted at 80°C for 4 hours, followed by post-curing at 120°C for an additional 4 hours. Once cured, the composite panel was removed from the mold and sent for water-jet cutting to achieve the desired specimen dimensions for mechanical testing, in accordance with the relevant standards.

Macroscopic images from mechanical tests:

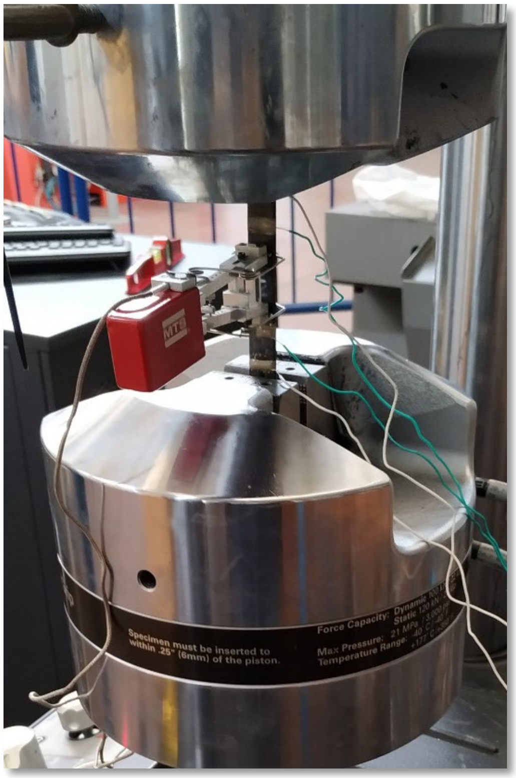

Figure S2 Experimental set-up of Tensile tests

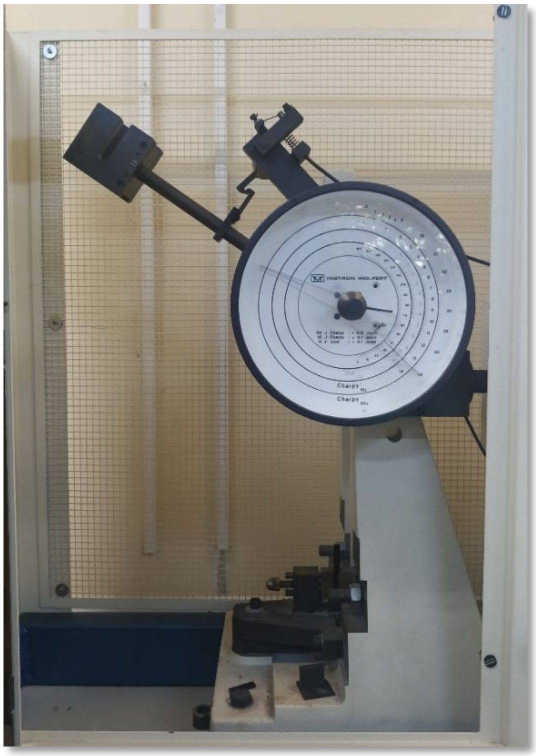

Figure S3 Experimental set-up of Impact tests

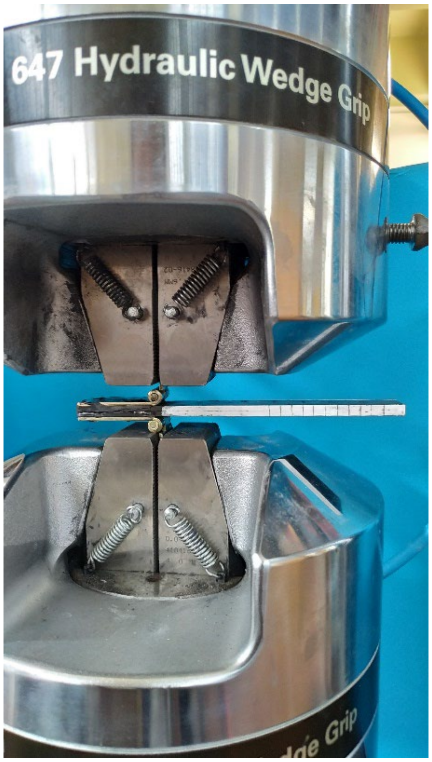

Figure S4 Experimental set-up of MODE I tests

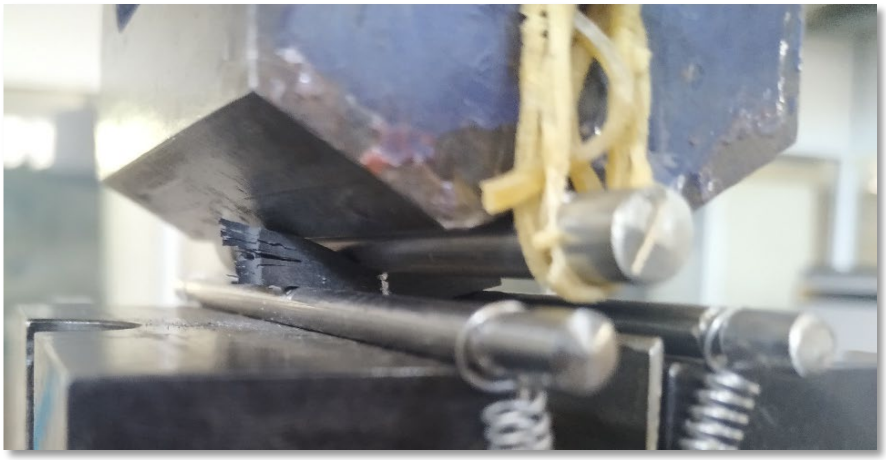

Figure S5 Experimental set-up of short-beam tests

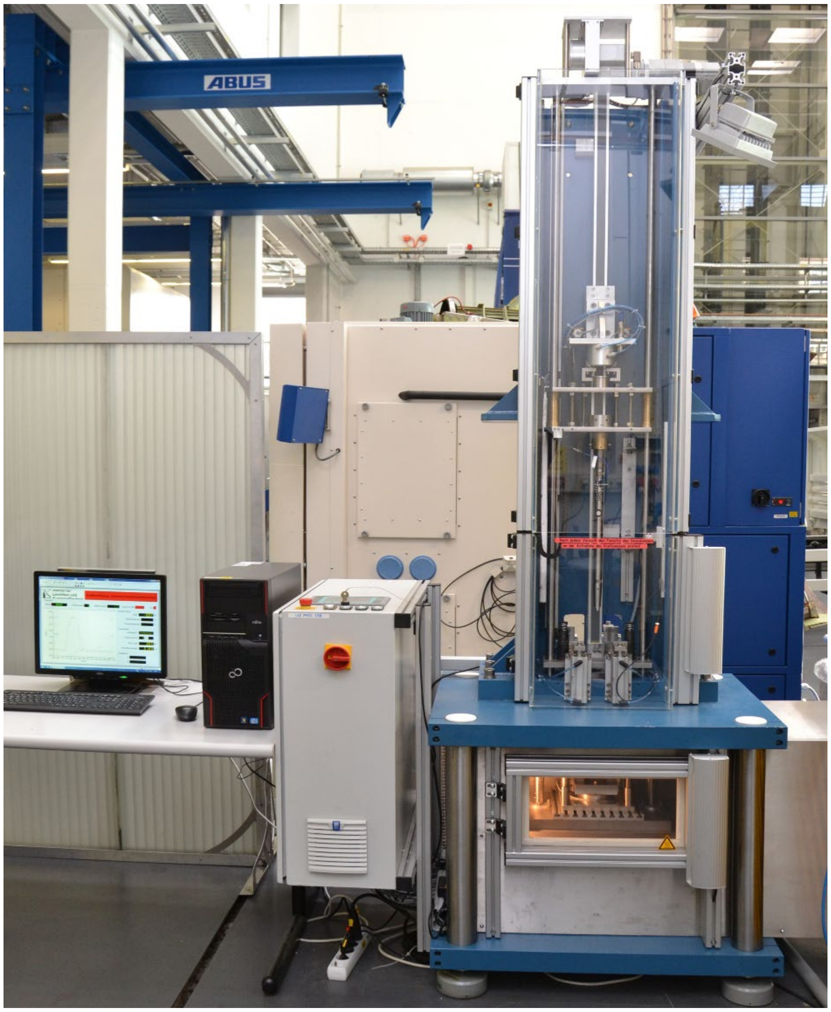

Figure S6 Experimental set-up of drop tower tests as performed in Institute of Lightweight Engineering and Polymer Technology, TUD Dresden University of Technology
